# Exploratory Analysis of Electronic Cigarette–Related Videos on YouTube: Observational Study

**DOI:** 10.2196/27302

**Published:** 2021-07-06

**Authors:** Zidian Xie, Xueting Wang, Yu Gu, Dongmei Li

**Affiliations:** 1 Department of Clinical & Translational Research University of Rochester Medical Center Rochester, NY United States; 2 Goergen Institute for Data Science University of Rochester Rochester, NY United States

**Keywords:** infodemiology, infoveillance, social listening, electronic cigarettes, e-cigarette, YouTube, user engagement, provaping, vaping-warning

## Abstract

**Background:**

Electronic cigarette (e-cigarette) use has become more popular than cigarette smoking, especially among youth. Social media platforms, including YouTube, are a popular means of sharing information about e-cigarette use (vaping).

**Objective:**

This study aimed to characterize the content and user engagement of e-cigarette–related YouTube videos.

**Methods:**

The top 400 YouTube search videos related to e-cigarettes were collected in January 2020. Among them, 340 valid videos were classified into provaping, vaping-warning, and neutral categories by hand coding. Additionally, the content of e-cigarette videos and their user engagement (including average views and likes) were analyzed and compared.

**Results:**

While provaping videos were dominant among e-cigarette–related YouTube videos from 2007 to 2017, vaping-warning videos started to emerge in 2013 and became dominant between 2018 and 2019. Compared to vaping-warning videos, provaping videos had higher average daily views (1077 vs 822) but lower average daily likes (12 vs 15). Among 161 provaping videos, videos on user demonstration (n=100, 62.11%) were dominant, and videos on comparison with smoking had the highest user engagement (2522 average daily views and 28 average daily likes). Conversely, among 141 vaping-warning videos, videos on potential health risks were the most popular topic (n=57, 40.42%) with the highest user engagement (1609 average daily views and 33 average daily likes).

**Conclusions:**

YouTube was dominated by provaping videos, with the majority of videos on user demonstrations before 2018. The vaping-warning videos became dominant between 2018 and 2019, with videos on potential health risks being the most popular topic. This study provides updated surveillance on e-cigarette–related YouTube videos and some important guidance on associated social media regulations.

## Introduction

Electronic cigarette (e-cigarette) use has increased significantly since its introduction in the US market in 2007, especially among youths [1,2]. Although the prevalence of e-cigarette use in youths decreased in 2020 compared to 2019, 19.6% of high school students and 4.7% of middle school students still reported using e-cigarettes [3,4]. The long-term health risks of e-cigarette use are still unclear; however, e-cigarette use has been associated with many health problems, including respiratory disorders [5-8], cardiovascular disease [9-11], and potential mental and cognitive problems [12,13].

e-Cigarettes are often marketed as healthier alternatives to cigarette smoking by e-cigarette companies on the internet [14,15]. People interested in e-cigarettes might seek further information about the product and its use on the internet, especially on social media [16,17]. Social media, such as Twitter, Instagram, and YouTube, have become a popular platform for e-cigarette users to share their experiences and for vaping companies to promote their products. Apparently, Twitter is dominated by tweets promoting e-cigarettes [18,19]; the e-cigarette–promoting posts have higher user engagement than e-cigarette–warning posts on Instagram [20]. Therefore, e-cigarette companies aggressively promote their products to the public, especially youth, through social media.

YouTube, created in 2005, is a popular social media platform with over 2 billion users and over billions of views daily [21]. YouTube was initially designed and created for sharing videos, but e-cigarette companies have used it extensively to promote tobacco products (including e-cigarettes) [22,23]. Many YouTube videos, especially on e-cigarettes, do not have an age restriction [24], making their promotional content on tobacco products easily accessible by youths, affecting their perception of tobacco products and causing major public health implications. As a result, YouTube has been promoting tobacco products, including e-cigarettes [25,26]; e-cigarette promotional videos were the dominant e-cigarette–related YouTube videos in 2012-2013 [24].

Social media such as YouTube has been widely used for sharing information and communicating with others. It is a rich data source for public health professionals to understand what e-cigarettes–related information is posted on YouTube and how they get disseminated, thus providing important information for public health surveillance. With the rapid increase of e-cigarette use in recent years, especially among youth, e-cigarette–related YouTube videos may have evolved. Therefore, it is important to examine more recent e-cigarette–related videos on YouTube to study the dynamic changes in such videos. Additionally, it is crucial to examine the user engagement of different e-cigarettes–related videos for some effective guidance on stopping the current vaping epidemic. This study aimed to characterize the e-cigarette–related YouTube videos by identifying provaping and vaping-warning videos and comparing their content and user engagement. Results from the study could help us understand what information related to e-cigarettes has been posted on YouTube and provide potential effective approaches to protect public health, especially among youths.

## Methods

### Data Collection

e-Cigarette–related videos and their associated metadata were downloaded from YouTube on January 12, 2020, with the search keyword “e-cigarette” using youtube-dl (a command-line program). Top search videos were selected based on their presence in the search, which might be more likely to be viewed by users. Out of the top 400 YouTube videos related to e-cigarettes, only 373 videos were successfully downloaded from YouTube. Among them, 20 videos were not in English, and 13 videos were uploaded after 2019. Finally, we obtained 340 unique e-cigarettes–related videos posted on YouTube between 2007 and 2019 and used them for further analysis.

We downloaded the metadata associated with each YouTube video, including video duration time (seconds), age limit, number of views, number of likes, and the posted date. Based on the posted date, we calculated the number of posted days for each video. To better compare the user engagement among different video categories, we normalized the number of views and likes to the number of posted days on YouTube for each video. The differences in user engagement measures (such as the number of likes and views) were tested by the two-sample *t* test at a significance level of .05 using the statistical analysis software R, version 4.0.3 (R Core Team).

### Video Hand Coding

To hand code the videos, 2 reviewers watched each downloaded e-cigarette–related YouTube video. The content of each video was summarized after watching it carefully. Each video was categorized as provaping, vaping-warning, or neutral based on its overall attitude toward e-cigarettes. A provaping video was defined as promoting e-cigarette use, such as showing certain e-cigarette products, vaping demonstrations, and the benefits of vaping. A vaping-warning video was defined as discouraging e-cigarette use, such as presenting the potential health risks of vaping and policies regulating e-cigarettes. Neutral videos did not clearly express either provaping or vaping-warning messages, such as explaining why e-cigarettes are popular or discussing their pros and cons.

Each video was categorized further based on its video content. Provaping videos were categorized as (1) user demonstration: showing how to use e-cigarettes and vaping tricks; (2) comparison with smoking: emphasizing that e-cigarettes were healthier and safer than smoking and can help quit smoking or replace cigarettes; (3) introduction of e-cigarettes: providing an introduction to e-cigarettes and available flavors; (4) reduced health risks: highlighting the reduced known (such as lung and respiratory) or unknown health problems; (5) product sale: including brand introduction and web links to purchase or obtain coupon; and (6) promoting e-cigarettes: underlining cost-savings, use of e-cigarettes anywhere, and the experience resembling that of real cigarettes.

Vaping-warning videos were categorized as (1) e-cigarette regulation: including vaping ban, legal fight against e-cigarettes, and the Food and Drug Administration (FDA) regulation; (2) comparison with smoking: arguing against healthier- and safer-than-smoking messages or unproven efficacy for quitting cigarettes; (3) potential health risks: showing known and unknown health risks, lung or blood problem, respiratory symptoms; (4) explosion hazard: describing accidents due to e-device explosion; and (5) youth addiction: including nicotine addiction in youth or role as a gateway drug.

The agreement between the 2 independent reviewers on attitude toward e-cigarettes was 87.43%, while the agreement on video content was 78.07%. Any discrepancy between the 2 reviewers was resolved by discussion among the 4-member research team.

## Results

### e-Cigarette–Related YouTube Videos

Among 340 e-cigarettes–related YouTube videos, 141 (41.5%) were vaping-warning videos, 161 (47.3%) were provaping videos, and 38 (11.2%) were neutral videos without any evident attitude toward e-cigarettes.

**Figure 1:**
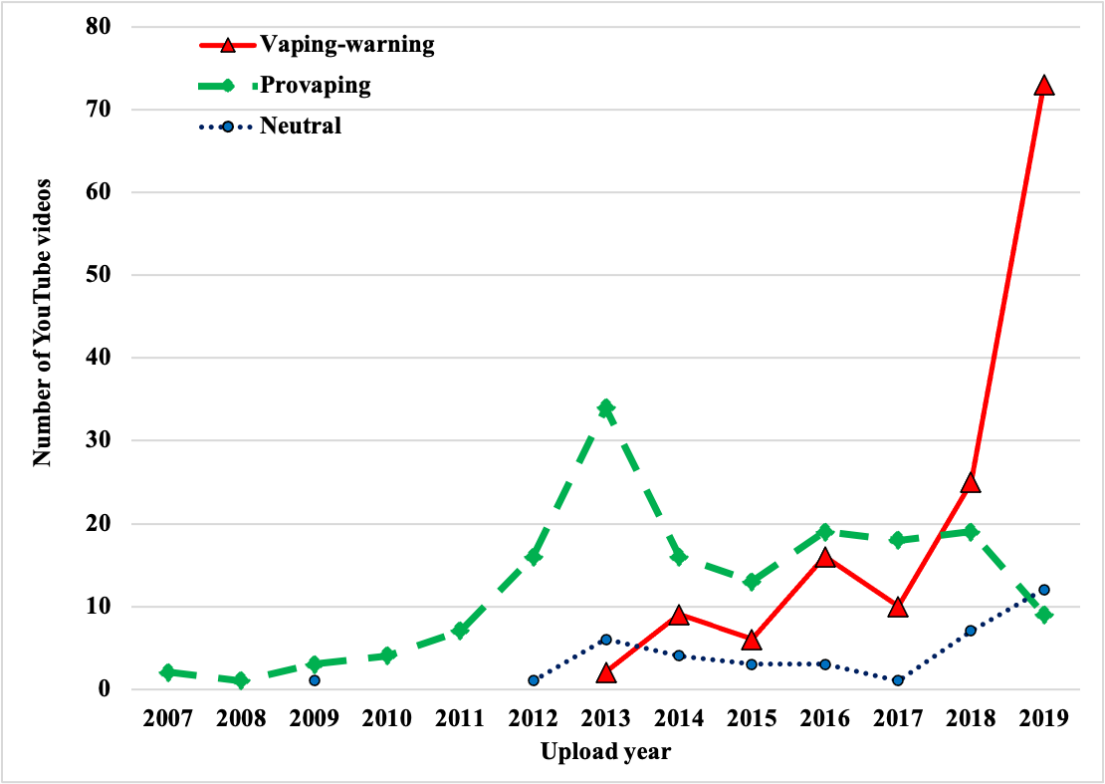
The popularity of e-cigarette videos on YouTube over time.

The videos shortlisted for the study were posted on YouTube between 2007 and 2019 (Figure 1). Beginning with 2007, the number of provaping videos kept increasing until 2013 and then significantly decreased in 2014. In contrast, the number of vaping-warning videos started increasing in 2013 and surged drastically in 2019. Neutral videos maintained a low level but exhibited a slight increase recently.

As shown in Table 1, we compared user engagement measures among different types of e-cigarette–related YouTube videos. Provaping videos were found to have a higher number of average views (778,569 vs 380,671, *P*=.24) and likes (7300 vs 5523, *P*=.70) than vaping-warning videos. Since the posted date for each video on YouTube was different (from 2007 to 2019), the number of days for each video posted on YouTube was also different. For example, the average number of posted days for provaping videos was 1806 days, whereas it was 605 days for vaping-warning videos. The average number of daily views for provaping videos was higher than that for vaping-warning videos (1077 vs 822, *P*=.72), but the average number of daily likes for provaping videos was lower than that for vaping-warning videos (12 vs 15, *P*=.85). The vaping-warning videos had a longer video duration (759.5 seconds vs 370.8 seconds) on average than provaping videos. The average number of daily views and likes for neutral videos was between the average daily views and likes for provaping and vaping-warning videos.

**Table 1:** Characteristics of e-cigarette–related YouTube videos.

Video categories	Videos, n	Views, mean (SE)	Likes, mean (SE)	Daily views, mean (SE)	Daily likes, mean (SE)	Posted days, mean (SE)	Video duration (seconds), mean (SE)
Vaping-warning	141	380,671 (166,961)	5523 (3444)	822 (527)	15 (12)	605 (55)	759.5 (134)
Neutral	38	251,231 (89,292)	2732 (1276)	506 (278)	14 (11)	1128 (159)	561.1 (159)
Provaping	161	778,569 (293,749)	7300 (3014)	1077 (493)	12 (6)	1806 (76)	370.8 (26)

### Provaping YouTube Videos

Among 161 provaping YouTube videos (Table 2), most (n=100, 62.11%) focused on user demonstration by showing how to use e-cigarettes and some vaping techniques, followed by product sale (n=25, 15.53%), and comparison with smoking (n=16, 9.94%). Among provaping videos, the videos that compared e-cigarettes with smoking had the highest number of views (2,121,410 views/video) and likes (21,512 likes/video) on average.

**Table 2:** Characteristics of provaping YouTube videos.

Topics	Videos, n (%)	Views, mean (SE)	Likes, mean (SE)	Daily views, mean (SE)	Daily likes, mean (SE)	Posted days, mean (SE)
Comparison with smoking	16 (9.94)	2,121,410 (978,736)	21,512 (9237)	2522 (1195)	28 (12)	1109 (214)
Introduction of e-cigarettes	8 (4.97)	225,026 (85,275)	3040 (1728)	165 (72)	3 (2)	1680 (427)
Reduced health risks	5 (3.11)	53,877 (38,799)	835 (726)	401 (386)	7 (7)	937 (441)
Product sale	25 (15.53)	96,232 (34,949)	686 (380)	113 (59)	1 (0)	1806 (154)
Promoting e-cigarettes	7 (4.35)	183,037 (128,176)	1429 (1237)	48 (29)	0 (0)	3260 (382)
User demonstration	100 (62.11)	856,504 (442,943)	7629 (4570)	1266 (767)	15 (10)	1869 (89)

Videos on user demonstration had the second highest number of views (856,504 views/video) and likes (7629 likes/video), followed by videos introducing e-cigarettes (225,026 views/video and 3040 likes/video) and promoting e-cigarettes (183,037 views/video and 1429 likes/video). After normalizing the number of views and likes to the number of posted days for each video, as shown in Table 2, the videos that compared e-cigarettes with smoking had the highest number of daily views (2522 daily views/video) and likes (28 daily likes/video), followed by user demonstration (1266 daily views/video and 15 daily likes/video), and reduced health risks (401 daily views/video and 7 daily likes/video).

### Vaping-Warning YouTube Videos

Among 141 vaping-warning YouTube videos, there were 57 (40.42%) videos on the potential health risks of e-cigarettes, 33 (23.40%) videos talking about e-cigarette regulation, and 25 (17.73%) videos showing exploded e-cigarette devices (Table 3).

**Table 3:** Characteristics of vaping-warning YouTube videos.

Topics	Videos, n (%)	Views, mean (SE)	Likes, mean (SE)	Daily views, mean (SE)	Daily likes, mean (SE)	Posted days, mean (SE)
Comparison with smoking	4 (2.84)	97,747 (71,649)	845 (638)	91 (35)	1 (0)	885 (425)
e-Cigarette regulation	33 (23.40)	35,853 (10,919)	566 (293)	256 (67)	3 (1)	173 (38)
Explosion hazard	25 (17.73)	326,335 (166,201)	1861 (1010)	243 (86)	1 (1)	1016 (113)
Potential health risks	57 (40.42)	595,728 (386,188)	10,802 (8400)	1609 (1299)	33 (29)	571 (88)
Youth addiction	22 (15.60)	453,889 (330,220)	4621 (3334)	423 (218)	4 (2)	823 (141)

Compared to other video types, videos about the potential health risks had a higher number of views (595,728 views/video) and likes (10,802 likes/video) on average. Videos showing youth addiction to e-cigarettes also had high views (453,889 views/video) and likes (4621 likes/video). After normalizing the number of days posted on YouTube, the videos about the potential health risks of e-cigarettes had the highest number of daily views (1609 daily views/video) and likes (33 daily likes/video), followed by videos on youth addiction (423 daily views/video and 4 daily likes/video).

## Discussion

### Principal Findings

In this study, we characterized the top 340 searches for e-cigarette–related videos on YouTube. While provaping videos were more prevalent before 2018, vaping-warning videos became dominant more recently (2018-2019). Additionally, the provaping videos had higher average daily views than the vaping-warning videos; the vaping-warning videos had relatively higher average daily likes than the provaping videos. Among the provaping videos, the majority were about user demonstration, which had relatively more daily views and likes on average than other video types except for the videos on comparison with smoking. Among the vaping-warning videos, the videos showing the potential health risks of e-cigarettes were the most prevalent, and they also had the most user engagement (daily views and likes).

Within the study time frame starting 2007, the number of provaping videos posted on YouTube increased continuously, reaching a peak in 2013, and then dropped significantly in 2014. In contrast, the number of vaping-warning videos recorded a continuous increase from 2013 and reached a significantly high level in 2019. On April 25, 2014, the FDA published a long-awaited proposed rule that put e-cigarettes under FDA regulation like other tobacco products [27]. Whether the FDA rule played a role in the dramatic decrease of provaping videos in 2014 needs further investigation in future studies.

Several studies have shown that YouTube has been used unevenly for promoting e-cigarettes in 2013 and 2014 [23,24,28,29]. In this study, we showed that provaping videos were dominant on YouTube before 2018, especially before 2014, which is consistent with the previous findings. An earlier study examined the total number of views for different e-cigarette–related YouTube videos; however, it did not calculate the average number of views for different types of videos and did not compare the user engagement among different videos [24]. This study examined the average number of views and likes for each type of video and calculated the average number of daily views and likes, reflecting user engagement on these videos. This study compared the three video categories and observed that the provaping videos had higher views and likes than the vaping-warning videos, consistent with the findings from a previous study [23]. Among provaping videos, about 10% of videos comparing e-cigarettes with smoking (mainly, vaping is a safer alternative to smoking) had the most user engagement (2522 daily views and 28 daily likes). Thus, there is some evidence that promoting e-cigarette as a safer alternative could influence its usage [30]. Furthermore, over 62% of provaping videos were about user demonstration and had a relatively high user engagement (average daily views and likes). Previous studies showed that product advertisement and user sharing related to e-cigarettes were the top genres on YouTube [23]. All e-cigarette–related YouTube videos collected in this study did not have an age restriction, suggesting that all YouTube users, including youth, could access these videos. Therefore, these provaping videos might have a great potential to promote e-cigarette use and might be partially responsible for the e-cigarette epidemic, especially among youth in recent years. Considering their prevalence on YouTube and high user engagement, these provaping videos should have an age verification system in place to prevent youth access.

Our study showed that among the 340 YouTube videos analyzed, the vaping-warning YouTube videos became dominant starting in 2018 and surged dramatically in 2019, correlating well with the EVALI (e-cigarette or vaping product use associated lung injury) epidemic in the United States [31]. Nearly half of YouTube videos in 2015 were educational, medical, or news videos [32]. Among vaping-warning videos, the videos on the potential health risks of e-cigarette use were the most prevalent and had the highest user engagement, providing a potentially effective way to inform the public, especially youth, about the health risks of e-cigarette use and protect public health. Therefore, these vaping-warning videos, especially those about the potential health risks of e-cigarette use, should be encouraged by public health authorities. The dominance of vaping-warning videos on YouTube in 2019 might contribute to the decrease of e-cigarette use, especially among youth in 2020 [3].

### Limitations

This study has several limitations. First, we collected only the top 400 searches for e-cigarette–related YouTube videos in the English language using the keyword “e-cigarette,” which might not represent the whole picture and introduce some potential biases in our results. In the future, other relevant keywords (such as “vaping” and “JUUL”) could be included to collect more e-cigarette–related YouTube videos for further analyses. Second, since there was no demographic information about the users (such as age and gender) available from YouTube, we did not know who watched these videos. Therefore, we could not determine the impact of these e-cigarette videos on different demographic groups. Third, this study’s relatively small sample size resulted in large variations and insignificant differences among different videos in terms of user engagement. Fourth, we did not segment each video in this study, possibly affecting the coding accuracy. Fifth, our content analysis was based on 340 videos only, and it is possible that there were other e-cigarette–related videos that could not be covered by our current categories. Sixth, this study examined the user engagement of each video type, which could be affected by the characteristics (number of subscribers and number of posted videos) of user accounts who posted these videos, especially the characteristics of those influencers. Therefore, it is important to understand how these features can affect the user engagement of e-cigarette–related videos in future studies. Finally, this study did not consider differences in geographic locations as YouTube is an international platform; however, different geographical locations and their impacts in different countries could be pursued in future studies.

### Conclusions

The study showed that e-cigarette–related YouTube videos were initially dominated by provaping videos and then vaping-warning videos, demonstrating the importance of such surveillance on YouTube to understand the dynamic changes in e-cigarette–related videos. Additionally, we showed different user engagement metrics for different e-cigarette videos on YouTube, providing important information for public health authorities to aid in developing appropriate regulations on social media to protect public health, especially among youth.

## Abbreviations

e-Cigarette: electronic cigarette
EVALI: e-cigarette or vaping product use associated lung injury
FDA: Food and Drug Administration
